# Recent Developments Relevant to Debates Around the Dissemination of Industry-Funded Science

**DOI:** 10.1093/ntr/ntae183

**Published:** 2024-07-20

**Authors:** Sophie Braznell

**Affiliations:** Tobacco Control Research Group, Department for Health, University of Bath, Bath, UK

Dear Editor,

I read with great interest the recent articles discussing the Society for Research on Nicotine & Tobacco’s (SRNT) policies around industry science and conflicts of interest^1,2^—an important issue in public health. I want to highlight recent developments of relevance to this debate.

In our latest paper published in *Nicotine and Tobacco Research* (NTR), my colleagues and I reported our examination of newly leaked documents from tobacco giant: Philip Morris International and its Japanese affiliate, Philip Morris Japan (PMJ).^3^ These documents laid bare PMJ’s work to use science and the scientific community to create favorable research and policy environments in which to sell its products, like IQOS. We found the activities described therein mirrored known strategies to manipulate science for profit, including attempts to influence the conduct, reporting, and reach of science, as well as efforts to conceal these activities.^3^

These findings build on our previous work and that of others, which has shown the tobacco industry’s science is often biased.^4–6^ The perils of biased science for public health are well known. It serves to muddy the water, making the potentially life-changing decisions of policymakers and the public harder.

Cummings et al.^1^ suggest decisions regarding the presentation or publication of research should primarily be based on scientific merit rather than funding source or affiliation. But the integrity of research extends beyond conduct and reporting; dissemination is equally vulnerable to bias arising from vested interests. Regardless of its scientific merit, the tobacco industry can (and does) misuse and misrepresent science to further its interests.^7^ Indeed, our examination revealed PMJ’s plans to leverage the company’s science and scientific reports from national government bodies to “build societal support for RRPs” (reduced risk products, which the company uses to refer to its newer nicotine and tobacco products like IQOS).^3^ Scientific merit is fundamental. However, even if the conduct and reporting were not at high risk of bias, publishing or presenting industry-affiliated research provides a platform from which it can misuse science and cultivate undue credibility.

The manipulation and misuse of science is not unique to PMJ. There is evidence of Philip Morris International replicating the activities we identified around the world.^8^ And it is not just the world’s biggest cigarette company. Evidence continues to emerge indicating that the wider tobacco and nicotine industry,^5,9,10^ as well as other industries,^7,9^ attempt to influence science to their benefit, often at the expense of public health.

Time and again the industry shows us it does not uphold the principles of open science, transparency or data sharing. In our examination, we noted lack of transparency was not limited to PMJ, but also the paid academics.^3^ Science from those who demonstrate their inability to uphold basic scientific principles should not be promoted. SRNT and NTR are to be commended for their commitment to this. Tobacco and nicotine companies looking to transparently share their research are still free to do so via their own websites, which could (and should) permit external review and comments.

As highlighted in our paper, it is clear that we are still in need of mechanisms through which the industry’s vast resources can be utilized to produce science while minimizing the bias arising from vested interests.^3^ In the meantime, I hope our recent findings are taken into consideration by SRNT, NTR, and other publishers when developing policies and procedures relating to the dissemination of industry-affiliated science.
